# Point-of-Care Ultrasound for Suspected Rib Fractures in Blunt Thoracic Trauma: A Proposed Emergency Department Protocol

**DOI:** 10.7759/cureus.117595

**Published:** 2026-10-07

**Authors:** Abdul Hamid

**Affiliations:** 1 General Practice, Royal Free Hospital, London, GBR

**Keywords:** chest radiography, chest wall trauma, clinical protocol, diagnostic yield, emergency ultrasonography, point-of-care ultrasound, rib fractures, thoracic trauma

## Abstract

Blunt thoracic trauma accounts for substantial emergency department admissions, with rib fractures representing a predominant injury pattern. Undetected or inadequately characterised rib fractures exacerbate the risks of hypoventilation, atelectasis, secondary pneumonia, and delayed pleuropulmonary complications, particularly in older adults. Conventional initial diagnostic evaluation relies heavily on plain chest radiography, which exhibits recognised diagnostic insensitivity for undisplaced cortical breaches and injuries traversing the costochondral junctions. Although computed tomography provides definitive anatomical resolution, routine acquisition in stable, minor blunt trauma imposes ionising radiation burdens, emergency department delays, and escalating healthcare expenditures. Point-of-care ultrasound (POCUS) offers an accessible, radiation-free imaging adjunct possessing high sensitivity for superficial cortical breaches, chondral disruptions, and adjacent pleural complications. To address persistent diagnostic blind spots and documentation deficits in acute thoracic care, a structured emergency department POCUS workflow is proposed. The operational protocol integrates targeted high-frequency linear interrogation of symptomatic cortical margins, multi-zone transducer exclusion of pleural collections and pneumothorax, standardised sonographic criteria, and a structured clinical escalation algorithm. When integrated with comprehensive clinical assessment, this proposed POCUS protocol is intended to support acute trauma stratification, facilitate targeted regional anaesthesia, optimise clinical documentation, and complement conventional radiography in acute care environments. Prospective implementation studies are required to determine its diagnostic accuracy and impact on patient outcomes.

## Introduction

Blunt chest wall trauma constitutes a major presentation to emergency departments, with low-energy blunt thoracic impacts precipitating more than half of all traumatic rib fractures [1]. The primary clinical presentation centres on debilitating thoracic pain exacerbated by deep inspiration and coughing, often resulting in prolonged physical disability lasting between two and eight months [2]. In acute settings, severe chest wall pain induces voluntary splinting and shallow respirations, initiating a pathological sequence of alveolar hypoventilation, progressive atelectasis, retained tracheobronchial secretions, and secondary pneumonia [3]. Acute complications encompass pneumothorax, haemothorax, pulmonary contusions, and flail chest architecture [3]. These clinical risks are heightened in older adults, where observed mortality in cohorts aged 65 years and older reaches approximately 19% [4]. Observational trauma registry data have identified the cumulative number of fractured ribs as an independent marker of injury severity, with a significant breakpoint in morbidity and mortality observed in patients sustaining six or more rib fractures [5]. Furthermore, the presence of three or more fractured ribs often signifies substantial blunt force transmission, serving as an established clinical threshold for hospital admission, multidisciplinary monitoring, or transfer to a specialised trauma centre [4,6]. Fractures traversing the first rib especially portend severe concomitant neurovascular or intrathoracic trauma [6].

The initial clinical evaluation in emergency triage has historically relied upon physical examination and standard plain chest radiography, yet both modalities harbour diagnostic limitations [7]. Physical examination frequently fails to reveal findings beyond localised thoracic tenderness, lacking the capacity to exclude bony discontinuity or occult parenchymal injury [7]. Plain chest radiography demonstrates a substantial false-negative rate ranging from 33% to 50% in observational series, routinely failing to detect undisplaced cortical breaches, fractures of the lateral rib arcs, and injuries traversing the cartilaginous costochondral junction [8]. While multi-detector computed tomography remains the reference standard for thoracic osseous and visceral imaging, routine acquisition for stable, minor blunt chest trauma exposes patients to considerable ionising radiation, creates workflow congestion, and increases emergency care expenditures [9]. Point-of-care ultrasound (POCUS) has emerged as an immediate, radiation-free imaging adjunct displaying superior sensitivity over radiography for superficial cortical and costochondral fractures [10,11]. Sonography also permits simultaneous bedside screening for secondary visceral complications, including traumatic pneumothorax and haemothorax [12,13]. Integrating bedside POCUS within acute trauma pathways is therefore intended to bridge the diagnostic gap between insensitive initial radiography and selective computed tomography in haemodynamically stable patients [11,14].

## Technical report

Standardised emergency department ultrasound protocol

Protocol Scope and Target Population

The proposed procedural protocol is intended as a structured bedside framework for haemodynamically stable adult patients presenting with focal chest wall tenderness following blunt thoracic trauma. It is designed to complement, rather than replace, standard trauma evaluation and clinically indicated imaging.

Equipment and Transducer Selection

The procedural protocol utilises two complementary transducers to achieve targeted thoracic assessment. Primary osseous interrogation utilises a high-frequency linear transducer (6-13 MHz) calibrated to a musculoskeletal or superficial preset, providing the axial and lateral resolution necessary to delineate the superficial hyperechoic cortical bone contour. Secondary visceral and pleural evaluation employs a low-frequency curvilinear or phased-array transducer (2-5 MHz) adjusted to a thoracic or abdominal preset, providing an acoustic penetration depth between 10 cm and 18 cm to evaluate for pleural sliding, lung pulse, parenchymal consolidation, and dependent pleural fluid.

Patient Positioning and Systematic Examination

The patient is ideally positioned in a semi-recumbent posture elevated at 30 to 45 degrees, or seated upright if haemodynamically stable, which maximises respiratory comfort and promotes gravitational accumulation of pleural fluid within the dependent costophrenic recesses. In immobilised patients or when spinal precautions are maintained, the examination proceeds in the supine position. The operator interrogates the symptomatic hemithorax along three longitudinal anatomical vectors: the anterior vector along the parasternal and midclavicular lines, the lateral vector across the anterior and posterior axillary margins, and the posterior vector traversing the scapular and paravertebral lines. The high-frequency linear transducer is placed perpendicular to the longitudinal axis of the rib in a transverse orientation to visualise adjacent rib acoustic shadows and intervening intercostal musculature, followed by the orthogonal rotation manoeuvre ninety degrees into a longitudinal plane aligned directly with the long axis of the rib. Interrogation commences directly over the epicentre of maximal palpation tenderness and extends systematically two rib levels superiorly and inferiorly. Anatomical regions that cannot be reliably visualised, such as posterior segments underlying the scapula (ribs one through three), should be documented explicitly as not assessed rather than assumed to be intact.

Diagnostic Criteria and Sonographic Findings

A suspected rib fracture is identified on longitudinal linear imaging by an abrupt cortical discontinuity, step-off deformity, or focal angular displacement interrupting the smooth, hyperechoic outer bony cortex [14,15]. Dynamic scanning during active respiratory excursions may accentuate fragment displacement or reveal movement between adjacent segments in undisplaced fractures. Posterior acoustic shadowing is noted deep to intact cortical margins. Fractures crossing the costochondral junction present as focal disruptions of the normally homogeneous hypoechoic cartilaginous matrix [8,10]. Associated soft-tissue signs include disruption of the overlying intercostal musculature and the accumulation of a localised, hypoechoic subpleural, periosteal, or intramuscular haematoma adjacent to the cortical breach [14,15]. Sonographic findings must be distinguished from normal costochondral transitions, prior consolidated fractures, or adjacent vascular grooves. Following osseous interrogation, pleural assessment is performed across anterior, lateral, and dependent posterolateral zones using the low-frequency transducer. The operator assesses for the presence of normal pleural sliding, B-lines, and lung pulse, while interrogating the dependent costophrenic recesses for anechoic or hypoechoic fluid indicating traumatic haemothorax [12,13]. A negative pleural examination does not exclude a small pneumothorax or anatomically occult collection.

Clinical Triage Algorithm and Escalation Framework

Diagnostic findings must be interpreted within a comprehensive clinical assessment incorporating patient age, frailty, pre-existing cardiopulmonary disease, analgesic requirements, inspiratory capacity, and mechanism of injury. In haemodynamically stable patients with isolated sonographic evidence of one or two undisplaced fractures, preserved bilateral lung sliding, absence of pleural fluid, and adequate pain control and ventilatory function, conservative outpatient management with multimodal analgesia and scheduled clinical review may be considered. Conversely, high-risk features warrant urgent multidisciplinary escalation and consideration of contrast-enhanced computed tomography in accordance with local trauma guidelines: the presence of three or more suspected fractured ribs, suspected fracture of the first or second rib, paradoxical segment motion concerning for flail chest, absence of lung sliding or identification of a lung point denoting traumatic pneumothorax, identification of pleural fluid, persistent hypoxaemia, or failure to control pain.

Standardised Reporting and Clinical Documentation

To promote clinical reproducibility and clear communication, every POCUS evaluation should be recorded using a standardised template within the electronic health record. The clinical record must capture the examination indication, probe types utilised, laterality, suspected anatomical rib levels, whether cortical breach or chondral disruption was observed, segments documented as not visualised due to anatomical obscuration, dynamic pleural sliding status across anterior and lateral zones, presence or absence of dependent pleural fluid, and any technical limitations encountered during the scan.

## Discussion

Emergency department evaluation of blunt chest wall trauma remains challenging due to the limitations of conventional physical examination and initial plain radiography for minor osseous trauma. Published diagnostic accuracy studies indicate that plain radiography misses 33-50% of rib fractures, frequently failing to identify cartilaginous and undisplaced lateral injuries [8,10,16]. By contrast, high-frequency linear ultrasonography has demonstrated high sensitivity for superficial cortical and chondral disruptions in prospective observational series, establishing its utility as an accessible bedside adjunct [10,11,17]. However, reported diagnostic sensitivity varies across the literature according to operator experience, scanning protocols, and the reference standard utilised (radiography versus computed tomography), and high sensitivity for superficial fractures does not negate the need for cross-sectional imaging when significant intrathoracic injury is suspected.

Failure to identify acute rib fractures carries significant physiological implications. Inadequately treated chest wall pain provokes voluntary respiratory splinting, predisposing vulnerable patients to rapid alveolar collapse, progressive atelectasis, and secondary bacterial pneumonia [2,3]. Displaced bony fragments also carry the potential to lacerate intercostal vessels or visceral pleura, contributing to delayed haemothorax or pneumothorax [3,13]. Because older trauma patients experience elevated morbidity and mortality following blunt chest trauma, rapid and accurate bedside identification of fracture-related pain is crucial for initiating proactive supportive care [4,5].

Standardised clinical documentation provides an essential framework for risk stratification and therapeutic planning. Identifying multi-level rib fractures or high-risk anatomical locations informs clinical admission thresholds and step-up trauma care [4,5,6]. Furthermore, contemporary regional anaesthesia modalities, including ultrasound-guided erector spinae plane blocks and serratus anterior plane blocks, depend upon accurate dermatomal mapping of injured segments to achieve effective somatic analgesia and preserve ventilatory function [18,19]. A synoptic electronic documentation template ensures that critical findings and technical limitations are clearly communicated across clinical teams. More broadly, standardised POCUS applications can provide rapid bedside decision support in time-sensitive emergency care, although diagnostic performance and workflow benefits remain application-specific [20].

Limitations and implementation considerations

Several clinical and technical limitations must be considered when implementing this proposed protocol. Ultrasound is an operator-dependent modality, and clinical uptake requires structured competency-based training, supervised scanning, image archiving, and ongoing quality assurance [11,16]. Examination feasibility and duration depend on patient body habitus, severe obesity, extensive subcutaneous emphysema, and pain-related intolerance during transducer application. Clinicians must recognise key anatomical blind spots: acoustic shadowing behind the scapula and clavicle routinely obscures the posterior aspects of the first through third ribs [10,11,17]. Crucially, POCUS cannot evaluate the mediastinum, exclude pulmonary contusion, or rule out deep vascular or diaphragmatic injuries. A negative or technically limited ultrasound examination must never override clinical suspicion or replace computed tomography when cross-sectional imaging is indicated by mechanism or physiological criteria. Prospective multicentre trials remain necessary to evaluate the diagnostic accuracy, interobserver agreement, and clinical outcomes of this structured workflow.

## Conclusions

This technical report outlines a proposed, standardised POCUS workflow for the evaluation of suspected rib fractures in haemodynamically stable adult patients with blunt thoracic trauma. By targeting symptomatic sites and providing systematic pleural screening, this structured bedside protocol is designed to complement initial plain radiography and clinical examination while facilitating precise documentation and targeted regional analgesia. POCUS should not be viewed as a replacement for computed tomography when major visceral injury is suspected. Prospective clinical studies are warranted to formally evaluate the feasibility, interobserver reliability, diagnostic performance, and impact on patient outcomes associated with the implementation of this proposed protocol.
